# Bronchobiliary fistula after ablation of hepatocellular carcinoma adjacent to the diaphragm: Case report and literature review

**DOI:** 10.1111/1759-7714.13380

**Published:** 2020-03-09

**Authors:** Zhi‐mei Huang, Meng‐xuan Zuo, Yang‐kui Gu, Chun‐xiao Lai, Qiu‐xiang Pan, Xiao‐cheng Yi, Tian‐qi Zhang, Jin‐hua Huang

**Affiliations:** ^1^ Department of Minimal invasive intervention, State Key Laboratory of Oncology in South China, Collaborative Innovation Center for Cancer Medicine Sun Yat‐sen University Cancer Center Guangzhou China; ^2^ Department of Gastroenterology Huangpu People's Hospital Zhongshan China; ^3^ Department of Medical Oncology TCM Hospital of Ruichang Ruichang China

**Keywords:** Ablation, bronchobiliary fistula, hepatocellular carcinoma

## Abstract

**Background:**

Bronchobiliary fistula is a rare, but life‐threatening complication after ablation of hepatocellular carcinoma. Few cases of bronchobiliary fistula have been reported and the treatment is controversial.

**Methods:**

From 2006 to 2019, a total of 11 patients were diagnosed with bronchobiliary fistula after ablation and received nonsurgical treatment.

**Results:**

All 11 patients presented with cough and bilioptysis. There were only two patients in which MRI revealed an obvious fistulous tract connecting the pleural effusion and biliary lesions. Pleural effusion, liver abscess and hepatic biloma were found in other patients. Three patients died of uncontrolled bronchobiliary fistula.

**Conclusions:**

Bronchobiliary fistula is a rare post‐ablation complication but should be taken into consideration in clinical decisions. Minimally invasive interventional treatment is a relatively effective means of dealing with bronchobiliary fistula, but as for the more severe cases, greater clinical experience is required.

## Introduction

Hepatocellular carcinoma (HCC), which originates from liver cells, is a common type of primary liver cancer and the second‐most frequent cause of tumor‐related mortality worldwide.^1^ Currently, ablation is commonly used as a powerful treatment for HCC. This minimally invasive and repeatable treatment has been shown to be similarly effective as surgical excision for the treatment of small liver cancers,^2^, ^3^ and in recent years, some medical centers have begun to apply ablation to the treatment of large HCC.^4^, ^5^

HCC ablation is associated with a low incidence of complications which are mostly mild‐to‐moderate, with reported overall complication rates of 2.2–5.7% and related mortality rates of 0%–1.4%. Notably, microwave ablation (MWA) contributes to higher mortality and complication rates, compared to radiofrequency ablation (RFA).^6^, ^7^, ^8^, ^9^, ^10^, ^11^, ^12^, ^13^, ^14^ Despite its minimally invasive and generally safe characteristics, ablation may also have a series of specific complications which should be considered when making clinical decisions, including hemorrhage, hepatic dysfunction, gastrointestinal injury, biliary tract injury, infection, pleural complications, bronchobiliary fistula, and vascular fistula.

Bronchobiliary fistula is a rare post‐ablation complication involving the formation of an abnormal channel between the biliary system and bronchial tree. The clinical manifestations of this complication include a recurrent bilioptysis, fever, jaundice, and chest pain. Some patients with a chronic course may develop potentially debilitating and life‐threatening conditions such as bronchiectasis, hepatic lung disease, and anemia.^15^ Relatively few published reports have reported bronchobiliary fistula after liver cancer ablation, and the treatment of bronchobiliary fistula are unclear. In this study, we retrospectively summarize the diagnosis and treatment of bronchobiliary fistula after HCC ablation at our center and analyze the possible pathogenesis of, and therapy for, bronchobiliary fistula based on the literature in order to provide clinical guidance.

## Methods

### Patient data

This retrospective study was approved by the Ethics Committee of our hospital, and all patients provided their signed informed consent before treatment. From May 2006 to August 2019, 1232 patients underwent computed tomography (CT)‐guided ablation of HCC at our medical center according to the Chinese primary liver cancer diagnostic and treatment practices (2017 edition).

There were 11 patients who suffered bronchobiliary fistula after ablation, with an average age of 51.5 years (range: 38–63 years), 10 of whom were male. Tumor lesions of five cases were located in hepatic segment seven and tumor lesions of six cases were located in hepatic segment eight. The average lesion diameter was 78.7 mm (range: 40–133 mm) and the average distance between the applicator and diaphragm was 3.6 mm (range: 2–5 mm). Microwave ablation was performed in 11 cases, and another two cases received radiofrequency ablation and cryoablation separately. Further details of the patients are summarized in Table 1.

**Table 1:** Clinical characteristics of 10 patients with bronchobiliary fistula

			Liver tumor			Ablation			Bronchobiliary fistula						Prognosis	
No.	Gender	Age	Location	Maximum diameter of tumor	Distance between applicator and diaphragm	Type	Time	Power/cycle	Time to ablation (days)	Symptoms	Imaging features	Infection	Management	Efficacy	State	Cause of death
1	Male	45	S7	97 mm	3 mm	MWA	12 minutes	65 W	7	Bilioptysis	Large amount of pleural effusion	No	Thoracic drainage	Unimproved	Dead	Related
2	Male	57	S7	50 mm	5 mm	MWA	12 minutes	65 W	30	Fever，Bilioptysis	Small amount of pleural effusion, liver abscess	*Escherichia coli* and fecal enterococci	Anti‐infection, Thoracic drainage	Improved	Dead	Unrelated
3	Male	63	S8	40 mm	3 mm	RFA	8 minutes	140 W	14	Bilioptysis	Small amount of pleural effusion, hepatic biloma	No	Biliary drainage	Improved	Dead	Unrelated
4	Male	58	S8	126 mm	5 mm	MWA	12 minutes	65 W	30	Bilioptysis	Medium amount of pleural effusion, hepatic biloma	No	Biliary drainage	Improved	Dead	Unrelated
5	Male	39	S8	40 mm	5 mm	MWA	10 minutes	70 W	8	Bilioptysis	Small amount of pleural effusion, hepatic biloma	No	Biliary drainage	Improved	Dead	Unrelated
6	Male	38	S7	133 mm	3 mm	MWA	10 minutes	65 W	15	Bilioptysis	Small amount of pleural effusion	No	Supportive treatment	Improved	Dead	Unrelated
7	Male	47	S7	70 mm	2 mm	MWA	8 minutes	60 W	14	Bilioptysis	Small amount of pleural effusion	No	Supportive treatment	Improved	Alive	‐
8	Male	62	S8	80 mm	3 mm	MWA	10 minutes	60 W	2	Fever，bilioptysis	Small amount of pleural effusion, liver abscess	Gram‐positive bacteria	Anti‐infection, Biliary drainage	Improved	Dead	Unrelated
9	Male	63	S8	100 mm	3 mm	Cryoablation	15 minutes	2 cycles	58	Fever，bilioptysis	Small amount of pleural effusion, liver abscess	Gram‐positive bacteria	Anti‐infection, Biliary drainage	Unimproved	Dead	Related
10	Female	46	S8	30 mm	3 mm	MWA	8 minutes	50 W	2	Fever，bilioptysis	Medium amount of pleural effusion	Fecal enterococci and *Klebsiella pneumoniae*	Anti‐infection, Thoracic drainage	Unimproved	Dead	Related
11	Male	49	S7	48 mm	4 mm	MWA	10 minutes	60 W	20	Bilioptysis	Small amount of pleural effusion, hepatic biloma	No	Biliary drainage	Improved	Alive	‐

### Ablation procedure

Under CT guidance (Elscint, Haifa, Israel), 1–5 applicators were placed percutaneously at the distal edge or interior of the tumor, depending on the tumor size. A single applicator was applied to tumors with a smaller diameter (<5 cm), while multiple applicators placed at intervals of <3 cm were applied to larger tumors. At least 5 mm adjacent normal liver tissue was ablated around the tumor to ensure a tumor‐free margin. If the lesion was near the diaphragm (distance ≤ 5 mm), the ablation was controlled to avoid direct damage to the diaphragmatic tissue. CT and CT/magnetic resonance imaging (MRI) were performed immediately postoperatively and after 3–6 months, respectively, to assess efficacy and complication.

## Results

### Characteristics of bronchobiliary fistula

In all 11 cases involving bronchobiliary fistula, the ablation for HCC was completed successfully, with no complication or secondary injury occurring during the operation. The patients developed bronchobiliary fistula‐related symptoms at an average of 19.8 days (range: 2–58 days) after the procedure.

**Figure 1:**
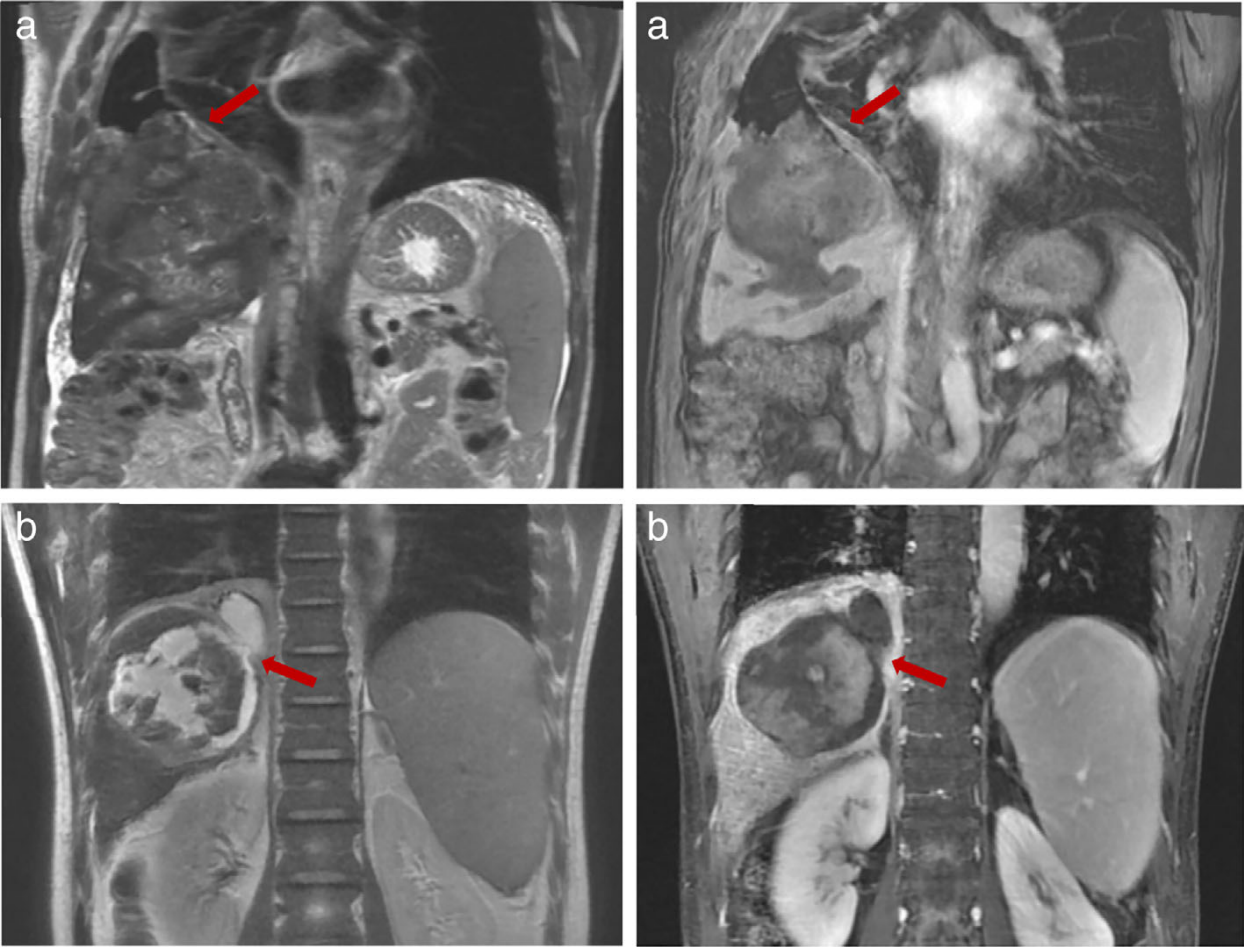
MRI image scans showing the fistulous tract connecting the pleural effusion and biliary lesions in two patients (a, b) with bronchobiliary fistula (red arrows).

**Figure 2:**
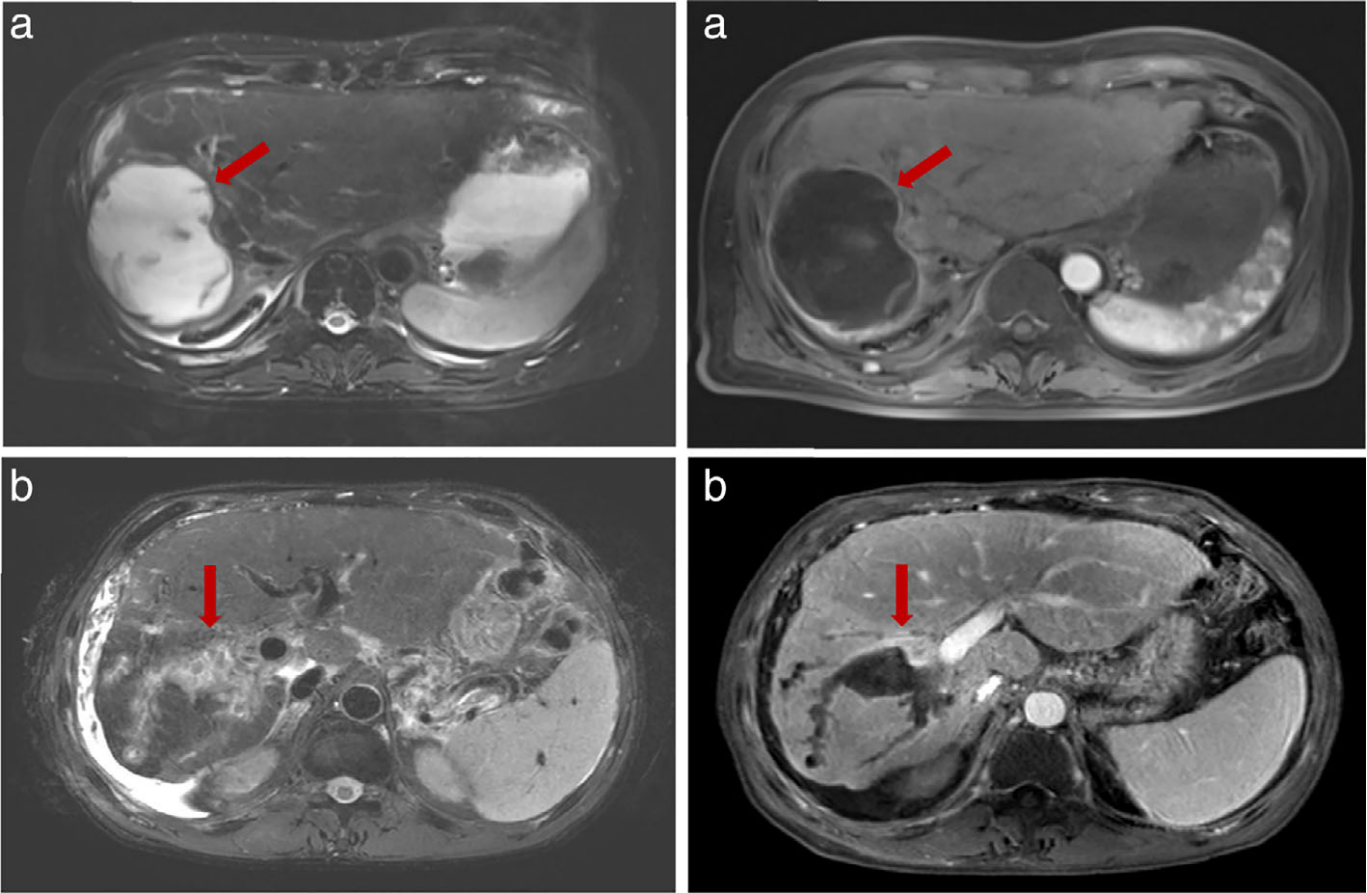
MRI image scans revealed biliary lesion in patients with bronchobiliary fistula. (**a**) Red arrow indicates the liver abscess after ablation. (**b**) Red arrow showed the biliary tract dilatation after ablation.

Diagnosis of bronchobiliary fistula principally relies on clinical symptoms and imaging. All 11 patients presented with cough and bilioptysis, which were accompanied with fever in four cases (Table 1). Imaging scans (CT or MRI) revealed that all patients had different amount of pleural effusion. There were 3/11 patients who developed liver abscess, while 4/11 patients suffered obvious hepatic biloma. However, an obvious fistulous tract connecting the pleural effusion and biliary lesions was revealed in only two patients on MRI imaging (Fig 1). Digital subtraction angiography (DSA)‐guided percutaneous transhepatic cholangiography (PTC) with a pigtail catheter was performed in the seven patients with obvious hepatic lesion, and injected contrast material revealed the presence of liver cavity or biliary dilatation. The remaining four patients exhibited unapparent or mild biliary tract dilatation (Fig 2).

*Escherichia coli* and fecal Enterococcus strains were isolated from a culture of the drainage material in one case, and in one case fecal Enterococcus and *Klebsiella pneumoniae* strains were isolated. Gram‐positive bacteria were found in two other febrile cases.

### Treatment and outcome

A total of seven patients consented to external biliary drainage as the main treatment for bronchobiliary fistula, of whom four patients manifested liver abscesses and were diagnosed as infection, so the anti‐infective treatments were applied simultaneously according to the pathological evidence. The symptoms of cough and fever immediately relieved in six patients after the initiation of drainage and anti‐infective treatment, and imaging examinations were repeated after continuous biliary drainage for a few months, which revealed satisfactory recovery of the liver and lung parenchyma, with no lesion between the intrahepatic biliary tree and bronchial tree. However, the treatment was poorly effective in one patient, and the patient finally died during follow‐up of an uncontrollable infection and liver failure due to inadequate bile drainage. Another five patients died of gastrointestinal bleeding or tumor progression.

Two patients presented with a small amount of pleural effusion were applied with supportive treatment under comprehensive consideration. After six months, the patients' discomfort resolved, and repeated imaging examinations revealed that the thoracic and biliary lesions had healed. One patient subsequently died of tumor progression, whereas the other achieved stable disease control and remains alive during the last follow‐up.

Although two patients had no significant biliary tract lesions, they complained of bilioptysis and presented with a medium to large amount of pleural effusion. Therapeutic percutaneous drainage was performed. After three days, the clinical symptoms of one patient disappeared, and the drainage volume decreased to <5 mL per day. Repeated imaging studies revealed a significant reduction in pleural effusion compared with the previous examination, and the drainage tube was removed. However, the patient immediately developed a significant pleural effusion with bilioptysis one week later and finally died of massive hemoptysis. Another patient simultaneously suffered infection and finally died of uncontrolled infection.

## Discussion

Any event that causes damage to the biliary tract, diaphragm, or bronchioles may contribute to a bronchobiliary fistula. This condition can be divided into congenital and acquired bronchobiliary fistula, the latter of which is mainly caused by primary or metastatic tumors.^15^ In recent years, the popularization and widespread application of ablation for the treatment of liver cancer^16^ has led to a gradual increase in the reports of postoperative bronchobiliary fistula. This specific type of iatrogenic bronchobiliary fistula is complex, associated with greater social problems, and places a greater burden on patients and their doctors. Therefore, it is very important to correctly diagnose and properly treat a postoperative bronchobiliary fistula.

Pathologically, postoperative bronchobiliary fistula arises from a biliary tract lesion after ablation. These lesions arise via two pathological mechanisms: (i) mechanic obstruction of the biliary tract by a thermal injury during treatment, and (ii) formation of a liver abscess or biloma after hepatic ablation.^17^, ^18^ In this study, three patients presented with obvious liver abscesses, whereas the others involved biloma or different degrees of biliary dilatation. These lesions can cause an increase in biliary or intracavitary pressure, which may drive bile or pus into the chest via the diaphragm due to negative pressure when combined with an injury of the diaphragm. The thermal injury during ablation is the most common type of diaphragmatic injury. All patients in this study who developed a bronchobiliary fistula had large liver tumors located near the diaphragm (the shortest distance was 2 mm). After ablation, CT/MRI revealed that, in all cases, the ablation borders reached the diaphragm and different degrees of pleural effusion had developed, indicating the thermal injury to the diaphragm during the ablation procedures and the subsequent formation of a bronchobillary fistula. Several case reports have also described the formation of bronchobiliary fistulae after the ablation of large tumors located near the diaphragm.^19^, ^20^, ^21^ The fistulae had a multifactorial etiology, and infection‐induced diaphragmatic injury can also contribute to formation of a bronchobiliary fistula. In this study, three patients presented with bacterial infections.

Bilioptysis, or a productive cough of bile‐stained sputum, is the most common and characteristic clinical manifestation of a bronchobiliary fistula. This manifestation can be detected through a biochemical examination. Other symptoms may include fever, chest pain, and abdominal pain.^15^ Some patients with emergent conditions may even develop an acute and life‐threatening lung or artery injury.^18^ All patients in this article presented with an expectoration of yellow, bile‐tinged sputum, and some patients also presented with a febrile infection.

Bronchobiliary fistula is mainly diagnosed with a CT or MRI scan. In this study, MRI only revealed obvious fistula tracts in two patients. By contrast, it is difficult to detect this tract in most cases, which is usually diagnosed with a combination of clinical symptoms and some indirect signs, including pleural effusion, atelectasis, liver abscess (cyst), and intrahepatic bile duct dilatation.^18^, ^19^, ^20^, ^21^, ^22^, ^23^ The invasive diagnostic tool PTC^24^ can be used under DSA guidance to visualize the entrance of contrast agents into the thoracic cavity or lung from an intrahepatic lesion. It also has a unique advantage that biliary or thoracic PTC drainage can be performed immediately.

Surgical and supportive therapy are generally applied for the treatment of bronchobiliary fistula,^25^, ^26^, ^27^, ^28^, ^29^ although the latter is only suitable for patients with mild symptoms. In our study, the fistula tract was not detectable and only accompanied by mild biliary dilatation in two patients, and these patients achieved relief with no recurrence after supportive treatment. However, supportive treatment does not effectively resolve the fistula in most patients, which often result in frequent relapse and eventual surgical intervention. Surgical treatments can be divided into open surgical and minimally invasive interventional treatments. The former includes conventional treatments for bronchobiliary fistula, including lobectomy, local hepatectomy, and diaphragmatic fistula repair.^25^, ^27^, ^28^ However, some patients are considered high‐risk for surgery, and resurgery after ablation is contraindicated for many other patients. In this study, 73% of patients underwent percutaneous transhepatic biliary drainage under CT or DSA guidance and received anti‐infective treatment while under full drainage. Only one patient died due to incomplete drainage, and the remaining cases resolved within six months, which is relatively effective and safe for patients with bronchobiliary fistula.

In conclusion, bronchobiliary fistulas are pathological communications between the biliary tract and the pleural space, which has a great effect on the treatment and outcome of HCC patients. Our study revealed that minimal invasive interventional treatment could be an effective and safety therapy for bronchobiliary fistula. However, when dealing with severe cases, more radical intervention may be necessary and further studies are required.

## Disclosure

The authors report no conflicts of interest.
